# Phase IV non-inferiority trials and additional claims of benefit

**DOI:** 10.1186/1471-2288-13-70

**Published:** 2013-05-30

**Authors:** Rosemarie DLC Bernabe, Grace Wangge, Mirjam J Knol, Olaf H Klungel, Johannes JM van Delden, Anthonius de Boer, Arno W Hoes, Jan AM Raaijmakers, Ghislaine JMW van Thiel

**Affiliations:** 1 Julius Center for Health Sciences and Primary Care, Utrecht University Medical Center, Utrecht, The Netherlands; 2 Division of Pharmacoepidemiology and Clinical Pharmacology, Utrecht Institute for Pharmaceutical Sciences, Utrecht University, Utrecht, The Netherlands; 3 National Institute for Public Health and the Environment, Ministry of Health, Welfare and Sport, Bilthoven, The Netherlands; 4 GlaxoSmithKline, Zeist, The Netherlands

**Keywords:** Non-inferiority, Post-authorization, Ethics, Additional benefit, Clinical relevance

## Abstract

**Background:**

Non-inferiority (NI) trials in drug research are used to demonstrate that a new treatment is not less effective than an active comparator. Since phase IV trials typically aim at informing a clinical decision, the value of a phase IV non-inferiority trial hinges also on its clinical relevance. In such trials, clinical relevance would refer to the added benefit claims of a specific drug, apart from efficacy, relative to its comparator drug in the trial.

**Methods:**

In this study, we reviewed 41 phase IV trials and extracted information on whether the authors mentioned any additional benefit beyond the NI (efficacy) claim of the drug and whether the additional benefit was proven in the trial. We checked whether the additional claim was based on descriptions only or on formal statistical analyses.

**Results:**

Our results showed that 22 out of the 41 NI trials mentioned additional benefit of the test drug and most of these claims were related to the safety profile. Of all the post-authorization NI trials that claimed additional benefit, 10 out of 22 NI trials used formal statistical analyses to show additional benefit, and only one included a sample size calculation for the additional benefit prior to the trial.

**Conclusion:**

We conclude that there is room for improvement in terms of designing phase IV NI trials with added benefit claims and in proving these additional claims.

## Background

Non-inferiority (NI) trials in drug research are used to demonstrate that a new treatment is not less *effective* than an active (i.e. effective) comparator [1,2]. Thus, an NI trial, which is mostly defined according to efficacy parameters, indirectly shows that the new treatment is also effective. However, the clinical significance of phase IV (i.e., “studies, other than routine surveillance, performed after drug approval and related to the approved indication” [3]) NI trials do not solely pertain to efficacy endpoints that were already established in pre-authorization trials. Rather, phase IV trials aim at “informing a decision” [4], or in ethics, such a trial should disturb equipoise, i.e., the “state of indifference or disagreement in the expert medical community about the net preferred medically established procedure” [5]. As such, in principle, all NI trials should aim at specifying and demonstrating additional benefit claims. NI trials performed after authorization have a reinforced obligation to make additional claims, apart from the primary (efficacy) endpoint, for the results of such trials to be clinically relevant. Such additional claims may relate to improved safety, but also optimization of the method of administration, improved compliance, and cost-effectiveness. Since the value of late stage NI trials depends on these additional claims, appropriate study design and/or tests to demonstrate scientific validity of such claims is truly important. Whether and how these claims are scientifically justified in the NI trials currently performed is, however, unknown.

In this study, we reviewed 41 published post-authorization NI trials and determined whether these trials reported benefit claims beyond clinical efficacy and how these additional claims were supported or proven in the trials.

## Methods

We included all post-authorization NI trial publications among the 232 publications used for our earlier review on NI trials [6]. In that review, we performed a search in PUBMED on 5^th^ February 2009, using the search terms, “non-inferior*”, “noninferior*” or “active control” and “equivalence”, in combination with the MeSH term “humans” and “Randomized Controlled Trial” as publication type. This search resulted in 669 articles and, based on pragmatic consideration rather than proper sample size calculations, we randomly selected 300 for our review. Subsequently, we excluded studies on bioequivalence, phase I studies, non-drugs trials, and articles that did not have full-text in English which resulted in 227 articles that reported 232 NI trials.

We extracted the phase of the trial according to statements in the publications or the referred clinical-trial database (e.g. clinicaltrials.gov). We could only identify the phase of 91 NI trials. Of the 91 trials, 15 were phase IV trials. For the remaining 141 NI trials, we compared the start date of the trial with the marketing approval date of the studied drug. The marketing approval dates were obtained from public domains. The first date of the marketing approval anywhere in the world was considered as the date of the drug’s approval. If the trial started later than the drug’s worldwide marketing approval date, we considered it a phase IV trial. Of these 141 NI trials, we identified 35 post-authorization trials. Hence, in total we found 50 post-authorization trials. We excluded trials that were aiming for the registration of a new indication (i.e., phase IIIB trials) by checking the aim of the trials stated in the article and by double-checking in the public domain via FDA and EMA websites. In total, we excluded nine phase IIIB trials. In the end, we included 41 phase IV NI trials in our analysis.

From each article, we extracted information on the type of drug, type of trial initiator, number of trial subjects, type of analysis (whether it is Intention-to-treat (ITT), Per-protocol (PP) or both) and the conclusion of the trial. We categorized the trials either as pharmaceutical-industry-initiated or non-pharmaceutical-industry-initiated. A trial is initiated by a pharmaceutical industry if besides the sponsoring there was active involvement of the pharmaceutical industry in the trial process. This involvement included any inputs of the pharmaceutical industry in writing the trial protocol, trial monitoring, data analysis, and reporting. If it is stated in the article that the pharmaceutical industry only gave unrestricted funding or grant, without any other involvement, we classified the trial as non-pharmaceutical industry-initiated.

Furthermore, we extracted information on whether the authors mentioned any additional benefit beyond the NI claim of the drug and whether the additional benefit was substantiated in the trial via descriptions (e.g., via simple distribution tables) or formal statistical analyses. We refer to a formal statistical analysis as the existence of a priori objective/hypothesis pertaining to the additional claim which was accompanied by a sample size calculation and (preferably) a data analysis plan. For example, if the author mentioned that the additional benefit of the new drug was its better safety profile, we evaluated whether the safety data were presented descriptively, or if any sample size calculation or any data analysis to establish statistical significance was reported to test the difference in safety profile between the two drugs. In addition, we extracted the authors’ conclusion on the additional benefit.

GW and RB extracted all data and, in case of discrepancies, reached consensus by discussion. All statistical analyses were performed using SPSS 19 (SPSS Inc, USA; http://www.spss.com).

## Results

### Description of the trials

Cardiovascular drugs and anti-infective drugs were the most frequently studied drugs (22% for each; Table 1). The majority of all the trials were initiated by the pharmaceutical industry (61%). In 73% of the NI trials, the tested drugs were concluded to be non-inferior to their comparators.

**Table 1:** Characteristics of the NI trials

	***N (%)***
	***(Unless stated otherwise)***
**I. Type of Drugs**	
Anti-infective	9 (22)
Cardiovascular system	9 (22)
Systemic hormonal preparations	5 (12)
Vaccines	5 (12)
Musculo-skeletal system	2 (5)
Nervous system	3 (7)
Antineoplastic	2 (5)
Others	6 (15)
**II. Type of trial initiators**	
Non-pharmaceutical industry	12 (29)
Pharmaceutical industry	25 (61)
Not clear	4 (10)
**III. Number of trial subjects (median (interquartile range))**	316 (196–629)
**IV. Type of analysis**	
Both ITT and PP	19 (46)
ITT only	13 (32)
PP only	8 (20)_
Unclear	1 (2)
**V. Conclusion of the trial**	
Non-inferiority	30 (73)
Superiority	2 (5)
Inferiority	6 (15)
Others	3 (7)
**VI. Mentioned additional benefit**	22 (54)

### Additional benefit

Of the 41 NI trials, 22 (54%) mentioned additional benefit of the test drug (Table 2). Among those 22 trials, the additional benefit of “better safety profile” was most often claimed (12 trials; 55%). Twelve trials (55%) stated that the claimed additional benefits of the test drug were proven in the current trial. In 10 trials (45%), formal tests were used to explore statistical significance of the claimed additional benefit, but only one performed a sample size calculation for the claimed additional benefit prior to the start of the trial [7].

**Table 2:** Characteristics of additional benefit claims

**Additional benefit**	**N**	**Presentation of additional benefit**		**Conclusion on additional benefit**		
**(N =22)**		**Statistical test**	**Descriptively**	**Proven**	**Not proven**	**Not explicitly discussed**
Convenient method of administration	1	0	0	0	0	1
Better safety profile	12	5	7	7	3	2
Better compliance	3	1	2	3	0	0
Less costly	1	0	1	0	0	1
Convenient method of administration and better safety profile	5	4	1	2	2	1

Of the 25 NI trials with pharmaceutical industry involvement, 14 (56%) mentioned additional benefit of the test drug, while among the 12 non-pharmaceutical industry initiated NI trials, five (42%) mentioned additional benefit of the test drug (Table 3). Fourteen of the 25 NI trials with industry involvement claimed several types of additional benefit; in five of these, statistical testing was performed, while eight simply discussed the additional benefit claims, and one did not discuss the additional benefit claim at all. For the five non-pharmaceutical industry initiated NI trials that claimed additional benefit, “better safety profile” was most often claimed (four trials). Four of the five latter trials used statistical tests to explore the additional benefit claim.

**Table 3:** Additional benefit claims based on types of sponsor

			**Additional benefit (row percentage)**					
		**Not mentioned**	**Convenient method of administration**	**Better safety profile**	**Better compliance**	**Less costly**	**Convenient method of administration and better safety profile**	**Convenient method of administration, better safety profile, better resistance profile**
**Type of initiators**	Non-pharmaceutical industry (n = 12)	7 (59)	0	4 (33)	0	0	0	1 (8)
	Pharmaceutical industry (n = 25)	11 (44)	1 (4)	8 (32)	1 (4)	1 (4)	3(12)	0
	Not clear (n = 4)	1(25)	0	0	2 (50)	0	1 (25)	0
**Conclusion of the trial**	Non-inferiority (n = 30)	15 (50)	1 (3)	8 (27)	3 (10)	0	2 (7)	1 (3)
	Superiority (n = 2)	0	0	2(100)	0	0	0	0
	Inferiority (n = 6)	2 (33)	0	1(17)	0	1(17)	2 (33)	0
	Other (n = 3)	2 (67)	0	1 (33)	0	0	0	0

## Discussion

In our study of 41 phase IV NI trials, 54% reported beneficial claims in addition to the NI claim and 55% of these claims were related to safety profile. Of all post-authorization NI trials that claimed additional benefit, 45% performed tests to show statistical significance, and only one included a pre-study sample size calculation for the additional claim.

In the introduction, we stated that a phase IV trial should aim at “informing a clinical decision.” We defined “informing a decision” to refer to clinically relevant differences that would allow physicians to reasonably choose one drug over another. As such, we have hinged our definition on the obligation of the physician to choose the best-suited therapy given the patient’s condition. However, these clinically relevant differences also matter in the decision-making processes of the other stakeholders such as the regulators, patient groups, pharmaceutical industry, and third party payers. The importance of these clinically relevant differences is illustrated by the emergence of relative effectiveness as an important issue in the post-authorization stage, especially for third party payers such as the health insurance agencies [4]. The European Commission’s High Level Pharmaceutical Forum defines relative effectiveness as “the extent to which an intervention does more good than harm compared to one or more intervention alternatives for achieving the desired results when provided under the usual circumstances of health care practice” [4,8]. Ultimately, the aim of relative effectiveness assessment is “to compare healthcare interventions in practice in order to classify them according to their practical therapeutic value” [8]. We can expect this issue to sharpen as drug registration moves towards a “live license approach,” i.e., an approach where launch is limited, and the widening of the scope of the license depends on post-authorization trial results [9]. In the latter case, relative effectiveness matters not only for the payers but also for the regulators. Clearly, pharmaceutical companies would need to demonstrate more than ever the added value of a new drug, or in our terms, they need to demonstrate clinically relevant differences.

Our results demonstrate that this need to establish clinically relevant differences in post-authorization NI trials through added benefit claims remains to be met. The issue is emphasized by the fact that among those that made additional benefit claims, only half used formal testing to establish statistical significance, and the other half merely presented their claims descriptively. It is questionable if it is acceptable to base decisions/judgments of clinical relevance if claims are not sufficiently supported by evidence, such as those trials that only provide descriptions of the additional benefit claims. Some may argue that some additional benefits, such as the convenience of an oral route of administration compared to that of the intravenous route, may be obvious; hence, there is no need for evidentiary support. However, even for such claims, evidence is needed, as patients’ preferences may be different. Oral route might be more convenient in the physician’s perspective, but for the patient, the shape or the taste of the pill may be real issues, and therefore, the intravenous route could be better.

Apart from these scientific and regulatory issues with post-authorization NI trials without added benefit claims, or those with added benefit claims but without (or with questionable) scientific evidence, there is also an issue with the ethical justification of these trials. It is ethical for a trial to begin with the assumption of equipoise with the aim of disturbing it. Equipoise justifies the inclusion of patient-participants since the state of equipoise retains the possibility of a medically endorsable therapeutic benefit. Disturbing equipoise unambiguously establishes the value of an intervention, and hence, a trial that aims to disturb equipoise also aims to “improve preventive, diagnostic and therapeutic interventions (methods, procedures and treatments)” [10]. A trial that does not show that intervention A is in some way better than intervention B does not contribute to the improvement of therapeutic interventions. Hence, a phase IV NI trial that does not aim to assess benefit claims of the new drug does not disturb nor is it expected to disturb equipoise precisely because its goal is simply to show that A is not worse than B, and not that A is in some way better than B, a goal that does not even partly resolve the state of indifference and/or disagreement in the expert medical community. As such, a phase IV NI trial without added benefit claims may have ethical justification issues. In our study, only half of the NI trials claimed such additional benefits.

Of the 25 pharmaceutical industry-initiated trials, about half (56%) claimed multiple additional benefits. The variety of additional benefit claims made by the industry seems encouraging, as this may be a sign of how the industry tries to resolve the relative effectiveness obstacle. However, the absence of statistical testing and the reliance on mere descriptions of the alleged benefit in majority of the pharmaceutical industry initiated post-authorization NI trials bring us back to the evidence-problem we discussed earlier.

Lastly, the limited (in terms of number and variety) additional benefit claims in NI trials from independent investigators and in government initiated trials may be an indication that non-industry bodies are still generally more concerned about the narrower concepts of safety and efficacy (as opposed to the wider benefit-risk assessment, which includes factors beyond safety and efficacy [11]). This is understandable and useful for regulatory purposes; but this situation does not help ease the impending relative efficacy and live license hurdles.

Based on the foregoing discussions, it is clear at this point that post-authorization NI trials need to be designed such that potentially, the resulting data are capable of disturbing equipoise and hence address issues such as relative effectiveness. This may be enhanced by closer and earlier collaboration between stakeholders [12,13]. In addition, in a previous article, we pointed out that NI trials may be designed in a manner that simultaneously shows the NI objective “with regard to drug efficacy and the objective of establishing superiority of the additional advantages of a drug over its active comparator.” [14] This may be a viable direction to follow if we are to revise the methodology of phase IV NI trials.

Our small sample size is a limitation of this study. In addition, clinical relevance cannot be directly investigated using our data, and as such, further research is needed. Another limitation will be the fact that our current analysis was part of previous analysis where we had randomly selected 300 articles from 669 articles found in Pubmed on the 5th February 2009. As expected, since that date, there were more articles on NI trials that were published and indexed in Pubmed. Thus, we missed recently published Phase IV NI trials. However, we do not believe the main message of our article, i.e., the importance of showing additional benefits in Phase IV NI trials, will differ much with addition of those new articles in our analysis. There may be concerns that phase IV trials could be described as superiority trials in PUBMED and the (subordinate) non-inferiority objective may not have been mentioned in the abstract. However, a trial is referred to as superiority or non-inferiority based on its primary objective and not on its secondary/subordinate objective. Hence, we believe we did not miss any phase IV NI trial given our search strategy. To include trials that are not explicitly stated as NI trials, in our opinion, would be extremely difficult. Our analysis was done based on published reports in PUBMED. We did not include trials databases, such as the clinicaltrials.gov, since data included in these databases are not suitable for our in-depth analysis (for example, data on NI margin and how it was determined are missing from such a database).

## Conclusion

Our study clearly shows that post-marketing NI trials vary considerably in their aims and claims. Importantly, only about half of the trials claimed additional benefit. Consequently, post-authorization NI trials need to be more robust, i.e., these trials must produce information that is directly useful to the clinical setting. Moreover, these trials must show scientific validity if they are to claim any additional value that physicians can bank on. Hence, there is room for improvement in terms of designing phase IV NI trials with additional benefit claims and in proving these additional claims.

## Competing interests

RDLCB’s and GW’s PhD projects were funded by the Dutch Top Institute Pharma. JAMR works and holds stocks in GlaxoSmithKline.

## Authors’ contributions

RDLCB and GW extracted data, analyzed the data, and drafted the entire manuscript. MJK and GJMWT double-checked the data, contributed new ideas, and went through the various drafts for revisions. JJMD, AB, and AWH contributed new ideas, and went through the various drafts for revisions. OHK and JAMR contributed new ideas and went through the final version. All authors read and approved the final manuscript.

## Pre-publication history

The pre-publication history for this paper can be accessed here:

http://www.biomedcentral.com/1471-2288/13/70/prepub
